# Syncope—Do We Need AI?

**DOI:** 10.3390/jpm13050740

**Published:** 2023-04-27

**Authors:** Brian Olshansky, Milena A. Gebska, Samuel L. Johnston

**Affiliations:** Division of Cardiovascular Medicine, Carver College of Medicine, University of Iowa, 200 Hawkins Drive, Iowa City, IA 52242, USA; milena-gebska@uiowa.edu (M.A.G.); samuel-johnston@uiowa.edu (S.L.J.)

Syncope is a form of transient loss of consciousness (TLOC) resulting from cerebral hypoperfusion and is characterized by rapid onset, short duration and spontaneous complete recovery [1,2]. Despite years of epidemiological evaluations, investigations, decision rules, evidence-based syncope calculators and diagnostic tools, the assessment of patients with syncope has not improved much over the past 40 years [3]. There is a substantial need to address this gap, but the next steps on how to evaluate and manage patients with syncope effectively remain obscure. How do we overcome this impasse?

The management of syncope is challenging for several reasons: (1) the characterization of the episode itself is difficult because syncope can mimic other conditions, including coma, intoxication, seizure or simple mechanical fall; (2) it is a common problem that occurs in all age groups and is often benign; (3) even an isolated episode of syncope may be a sentinel premonitory event forecasting multiple recurrences, imminent sudden cardiac death or long-term risk of cardiovascular and total mortality, whereby proper intervention could improve outcomes; (4) the crux of the evaluation of syncope is in its history, yet the accuracy of gathering and documenting meticulous historical details related to a loss of consciousness is difficult in contemporary clinical practice; (5) diagnostic evaluations, which are often elaborate and expensive, can remain misdirected and fruitless and (6) the long-term management of syncope can be uncertain, even under the best of circumstances.

Artificial intelligence (AI) has begun to revolutionize medicine. Could AI be the next step in the management of patients with syncope? Surprisingly, despite the magnitude of the problem, as well as recent technological advancements in AI, there has been a paucity of literature on the subject. Is it reasonable to expect that AI will begin to help us to manage patients with syncope? Not yet.

However, we have already shown that AI can help to predict the length of hospital stay in patients who attend the emergency department after experiencing syncope [4]. AI excels at analyzing large datasets and can identify non-intuitive, subclinical and obscure patterns without the need to understand biological mechanisms. Within the problem of syncope, an ideal tool would streamline the patient’s evaluation, eliminate unnecessary testing and therapy, reduce unnecessary hospitalizations and stratify risk.

Is this possible? AI must begin somewhere. Just like a novice medical student during early clinical experiences, AI can learn from the expertise of supervising clinicians too, but on a much larger scale. Unlike traditional computer-based models that follow rules set by humans, AI can provide analyses by creating their own algorithms or learning from specific examples. For machine learning (ML) to be effective in the evaluation and management of patients with syncope, it will need to learn from robust granular clinical examples and datasets, including scanning patient charts using natural language processing (NLP) and performing graphical analyses of various clinical imaging modalities. Such datasets may need to be created and adjudicated by human experts [5]. The initial efforts will likely depend on supervised learning models, in which AI is taught and trained using reliable data and overseen by syncope experts. The role of a supervised learning approach may be limited. Well-trained, experienced physicians, even with reliable data and the latest guidelines, often remain puzzled and at an impasse with regard to syncope.

Unsupervised learning may be where the greatest potential of AI in relation to syncope lies. Unsupervised learning approaches can be performed without labels, but with clustering and thus learning without a teacher. This “black box” approach utilizes metadata that arrives at conclusions or decisions without explanation. New approaches may be discovered including risk assessment based on genomics, or other yet-known features.

AI could become a powerful tool for clinicians, rather than a replacement. Over time, AI may create prediction models regarding prognosis and recurrence. Compared to the standard contemporary practice of applying generalized national guidelines, AI may be more nuanced. It may provide a directed and personalized approach based on unique clinical characteristics and the specifics of history derived from NLP. AI may incorporate data from diagnostic tools, such as imaging studies, laboratory values and the electrocardiogram, in a way that has been presently unforeseen. Furthermore, AI may be able to integrate laboratory-based findings with clinical history and patient characteristics in a way that even the most expert clinician is not capable of achieving.

Proper management strategies utilizing AI may require a joint effort with a multidisciplinary expert panel to define “the truth” regarding an individual patient. After implementation, and after defining clinical endpoints, AI-augmented care could be compared with the standard-of-care practice offered by experienced, well-trained clinicians in a randomized controlled clinical trial. The goal would be to see whether machines, functioning as “superhuman assistants”, improve care.

Ultimately, AI may help to manage syncope independently and/or facilitate provider care at multiple levels to improve healthcare delivery, triaging and management strategies. Challenges in achieving this may be substantial and include poor documentation and the inadequate quality of large datasets, thus confounding predictions. Also, mistakes can be made. Can we trust AI when it comes to *primum non nocere*? This is uncertain. Who is responsible if AI goes wrong? This is also uncertain, but must include the clinician who has utilized AI in clinical decision-making [6].

It may turn out that AI is simply not capable of facilitating the care of the syncope patient. Syncope may be too complex, even for AI. Can AI augment or even replace the present science and art of medicine when it comes to syncope? The only way to determine this is to test its capabilities.

Given the lack of progress in the management of patients with syncope, we should embrace the possibility of applying AI/ML to the challenges inherent in syncope care. Although the consequences are unforeseen, the real goal of optimizing patient care is sacrosanct. AI, at its rather nascent stage, may be limited. Ultimately, artificial super-intelligence might exceed our expectations and surpass capabilities beyond what we can presently imagine.

We have reached an impasse in the management of syncope. Years have passed without new breakthroughs. So, where do we go from here? AI may be the next step. We need to define where and how AI can be implemented to improve the diagnostic strategies and outcomes in patients with a loss of consciousness that is thought to be due to syncope. We need to determine whether AI is feasible, reliable, and free of ethical bias. Once implemented, we will require prospective validation studies from the initial data analyzed retrospectively. Multicenter studies will be required to determine whether the information is robust and applicable to all patients. Such international high-quality datasets and clinically relevant ML models will require close collaboration between multidisciplinary syncope diagnosticians, AI experts, data scientists and medicolegal experts.

AI, indeed, may be the next step in guiding physicians in syncope care. The journey is only beginning. It is our hope that we are heading in the right direction.
